# Eddy Current Sensor Probe Design for Subsurface Defect Detection in Additive Manufacturing

**DOI:** 10.3390/s24165355

**Published:** 2024-08-19

**Authors:** Heba E. Farag, Mir Behrad Khamesee, Ehsan Toyserkani

**Affiliations:** Department of Mechanical and Mechatronics Engineering, University of Waterloo, Waterloo, ON N2L 3G1, Canada

**Keywords:** eddy current, non-destructive testing, probe design, additive manufacturing

## Abstract

Pore and crack formation in parts produced by additive manufacturing (AM) processes, such as laser powder bed fusion, is one of the issues associated with AM technology. Surface and subsurface cracks and pores are induced during the printing process, undermining the printed part durability. In-situ detection of defects will enable the real-time or intermittent control of the process, resulting in higher product quality. In this paper, a new eddy current-based probe design is proposed to detect these defects in parts with various defects that mimic pores and cracks in additively manufactured parts. Electromagnetic finite element analyses were carried out to optimize the probe geometry, followed by fabricating a prototype. Artificial defects were seeded in stainless steel plates to assess the feasibility of detecting various flaws with different widths and lengths. The smallest defect detected had a 0.17 mm radius for blind holes and a 0.43 mm notch with a 5 mm length. All the defects were 0.5 mm from the surface, and the probe was placed on the back surface of the defects. The surface roughness of the tested samples was less than 2 µm. The results show promise for detecting defects, indicating a potential application in AM.

## 1. Introduction

Non-destructive testing (NDT) is a method of testing materials for damage without harming the object under test [1]. There are two NDT categories. A category in which a good contact between the sensor and the surface of the part under test is required, such as eddy current testing, magnetic testing, ultrasonic testing, and electromagnetic testing. The second category does not require contact between the sensor and the surface of the part under test, such as thermography [2], radiography testing, and visual inspection [3]. Non-destructive testing is used as a quality assurance measure in many technologies, such as additive manufacturing. Additive manufacturing (AM) is the process through which objects are created from a three-dimensional, computer-aided design dataset; the parts are created by laying materials layer by layer [4]. There are seven categories of technologies for AM. The categories are powder bed fusion, material jetting, material extrusion, binder jetting, vat photo-polymerization, sheet lamination, and directed energy deposition [5].

**Figure 1:**
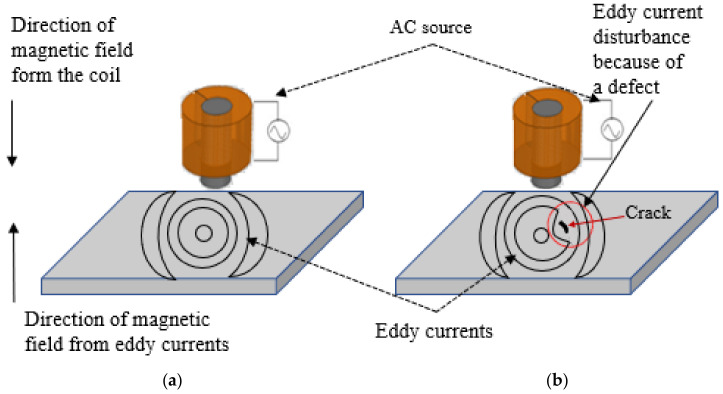
Distribution of eddy currents under the coil: (**a**) shows the eddy current distribution in a specimen without a defect inside and (**b**) shows the disturbance of the eddy currents in a specimen because of a defect inside it.

AM is used in many applications and fields such as aerospace for building components such as rocket engines. It is also used in automotive production to build lightweight components that lead to more efficient vehicles. In the medical field, it is used to fabricate replacements for damaged human tissues in the biomedical field [4]. Because of the wide range of applications for the additively manufactured parts, NDT techniques are required for inspecting these parts. The laser additive manufacturing process (LAM) involves many parameters which affect the quality of the produced parts. Defects such as pores and cracks are created within the parts because of the unmelted powder or excessive material vaporization during laser–material interaction. It is required to examine additive manufacturing parts to ensure the product’s integrity, quality, and reliability [4]. NDT techniques such as thermography, acoustic emission testing [6], ultrasonic testing, visual inspection, magnetic particle testing, radiography, and eddy current testing are used as in-situ and post-process inspection methods. Each one of these techniques has its advantages and disadvantages. In this paper, the focus will be on the eddy current NDT technique. Eddy current is based on the electromagnetic induction principle. In case of subjecting a part made of metal to a varying electromagnetic field, that is created by passing an alternating current through a coil, eddy currents will be produced inside that metal part. When the coil is placed over a conductive material, the eddy current flows in the test piece in a circular path or a loop as shown in Figure 1. The loop currents induced in the material produce an additional magnetic field. The magnetic field produced by the eddy currents opposes the magnetic field of the testing probe (Lenz’s law) [7,8]. A sensor can be used to measure the total magnetic field near the test part [7]. The existence of any cracks or defects perturbs the distribution of the eddy current’s field, which causes variations in the phase and magnitude of it. A receiver coil can be used to monitor these variations.

Eddy current is used in many different applications, such as inspection of the heat exchanger, inspection of bolt holes, pipe inspection in power plants, and inspection in high-speed rails. Also, the eddy current technique is used for inspecting parts produced by additive manufacturing technology, such as parts produced by powder bed fusion. For example, in directed energy deposition processes, pores are created by the release of gas during melting because of the moisture in the powder. In addition, the lack of powder fusion due to using an inadequate energy density may result in forming defects at the layer interfaces. Also, because of the solidification process, residual stresses are generated, and cracks are created in the produced parts [9]. In this study, the effectiveness of the eddy current technique is investigated to detect small subsurface defects such as pores and cracks in the range of 0.3 mm to 0.6 mm in size for either notch type, which simulate cracks, blind holes, and pores. Crucial aspects of the EC testing technique include how to generate the eddy currents to penetrate the part under test, and how to detect the signal that indicates the existence of a defects. These aspects make probes one of the most important components for this technique. In EC testing, there are different probe designs that are used for defect detection in different applications. One of the basic eddy current probes used for testing has one cylindrical coil which is used simultaneously to induce eddy currents and sense the defect in the metallic part.

**Figure 2:**
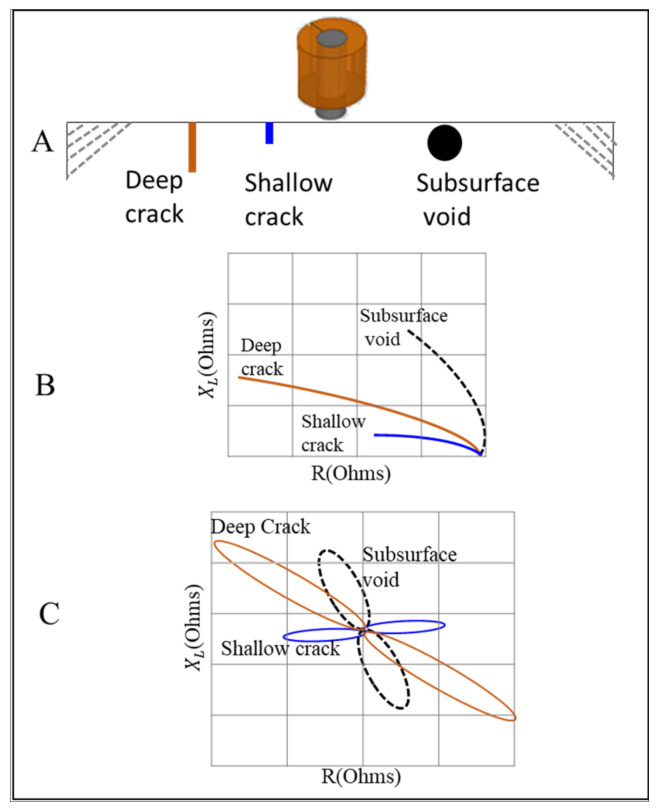
Probe signal representation of defects at different depths: (**A**) shows the probe on top of material with different defect types, (**B**) shows the EC signal representation of each defect from an absolute probe, and (**C**) shows the EC signal representation from a differential probe.

To determine how deep the eddy currents can go through the material, the depth of penetration formula given in Equation (1) can be used. It depends on the material conductivity and permeability of the test piece and the frequency used for the alternating current source.

(1) $$\delta \approx \frac{1}{\sqrt{\pi f\mu \sigma }}$$

where, δ is the standard depth of penetration (SDP), *f* is the frequency (Hz) during the test process, σ is the material conductivity, and μ is the material magnetic permeability of the part under test [10]. Usually, for a high depth of penetration, low frequencies are preferred in the case of subsurface defects. In the case of surface defects, high frequency is suitable to be used since there is no need for a high depth of penetration. The SDP only works in conditions that do not happen in reality; thus the frequency range can be tuned for the flaw and probe being used. Because of the skin depth effect the density of the eddy currents decreases with the depth. Phase lag *β*, given in Equation (2), means that the subsurface eddy currents lag at the surface and not in phase

(2) $$\beta =\frac{\chi }{\delta }$$

where (*χ*) is depth under the surface and (δ) is the SDP shown in Equation (1). Phase lag varies with the test frequency since it is a function of the standard depth of penetration. It allows for distinguishing between which signals represent an actual defect and which are irrelevant indications. Figure 2 shows that the phase angle representation of the subsurface void is higher than the ones obtained from the deep crack signal on a plate made of aluminum, although the crack goes deeper inside the plate. This is because of the exponential attenuation in EC density under the surface, which causes the contribution of the crack’s top half to the integrated signal to be higher than the contribution of the bottom one [11].

One of the challenges in detecting void flaws in-situ is the surface roughness. Surface roughness can be caused by either the solidification of the melt pool or by adhered powder particles. Some factors such as alloy uniformity and surface roughness can affect the detected eddy current signal. When testing metallic components, bad surface condition is a concern because it limits the possibility to detect surface defects. This is because rough surfaces produce very similar signals to the ones detected from the shallow surface defects of the part under test. As a result, the accuracy of detecting actual shallow surface defects will be low. Also, any changes in the electrical conductivity or magnetic properties of the material will affect the eddy current response [11]. For example, the alloy segregation that casts and welds often have will cause resistivity and magnetic permeability variations, leading to the formation of defects. Also, it will lower the signal-to-noise ratio (SNR), which will reduce the sensitivity and accuracy of detecting defects. The depth to which inspection can be carried out will have a big impact on the size of flaw that could be reasonably detected. Eddy current is dispersive, losing signal strength exponentially with increasing depth while at the same time increasing the area over which the current field is spread. The result is that the deeper the current goes into a material the larger in area the flaws need to be in order to maintain detectability. To achieve greater depths of penetration, a lower test frequency should be selected while using larger diameter coils or coil pairs with greater separation, exacerbating the need for larger area flaws. Another factor that affects the accuracy of the detected defect signal is the noise level. Since the ECT signal is small, noise is always a concern.

It is important to figure out where the noise is coming from and determine if the noise is coming from outside sources or within the ECT system itself. The design of the grounding and shielding scheme is very important as this significantly affects the noise levels of the system. In this paper, the design and implementation of a probe to detect subsurface pores and cracks in additively manufactured parts is shown in Section 2. Testing of the designed probe on stainless steel samples with different defect sizes is shown in Section 3. The samples are made of stainless steel (SS316L) by laser powder bed fusion (LPBF) technology. Testing was carried out on the opposite side of the defect to simulate subsurface defects. Finally, conclusions and proposals for future work are shown in Section 4. To avoid noisy or rippled signals resulting from the rough surface, the probe’s bottom surface was encapsulated with a coating layer to smooth out the detected signal from any ripples. The probe was moved across each defect multiple times to get the average signal value and standard deviation for each defect for more accurate results. A total of six readings were taken for each defect. The lift-off distance between the probe and the sample was kept constant by moving the probe back and forth on top of each defect without lifting it up.

## 2. Design and Implementation

### 2.1. Overview

One of the important factors that enables the probe to be sensitive to small defects is the coil dimension. A coil’s diameter becomes wider as its operating frequency lowers (i.e., a 1 mm diameter coil will not work at 1 kHz). Therefore, for eddy currents to find defects in 2 mm of aluminum, the test frequency needs to be low, hence a larger diameter coil is required. The minimal detectable flaw size is proportional to the diameter of the coil. As coil diameter increases, the minimum detectable flaw also increases. For notches, the rule of thumb is a quarter of a coil diameter. No matter what, the smaller the coil diameter, the smaller defect it can find. There are a lot of games to play to optimize a coil’s design, such as coil geometry, shielding, the use of ferrite cores, the number of turns, and a cross-section of wire.

### 2.2. Probe Design and Coil Dimension Determination

**Figure 3:**
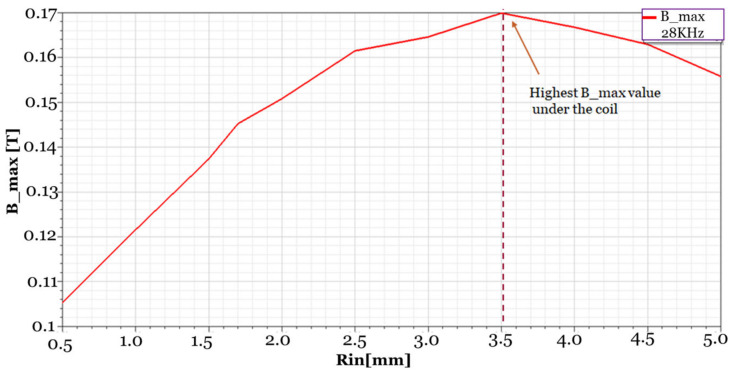
Sweep over the inner radius of the coil, the data exported from ANSYS maxwell, and the best B_max value is at inner radius 3.5 mm.

To be able to determine the best dimensions for the coil design, such as inner diameter, outer diameter, and length, a parametric sweep was carried out using ANSYS Maxwell over the coil’s inner radius for values from 0.5 mm to 5 mm. The outer radius value was fixed at 8 mm to get the best value for the magnetic field at a distance under the coil with respect to the inner radius at 28 kHz. In Figure 3, the best value of B_max is at an inner radius value (Rin) equal to 3.5 mm. The mesh type used for the simulation was an on-selection length-based type. The element length specified for the mesh was applied for the range of 3 mm to 16 mm.

After determining the inner and outer diameter of the coil, the next step was to determine the length of the coil. Using the Fabry factor formula in Equation (3) to get the best coil length, which gives the highest value of the B_max, helped to determine the last important coil dimensions for the probe design.

(3) $$G(\alpha ,\beta )=\frac{\sqrt{2\pi }}{5}\sqrt{\frac{\beta }{\alpha^{2}-1}}\;\ln(\frac{\alpha +\sqrt{\alpha^{2}+\beta^{2}}}{1+\sqrt{1+\beta^{2}}})$$

where, *α* = *D/Di*, *β* = *L/Di*, coil length is *L*, *D* is coil outer diameter, and *Di* is coil inner diameter. When the Fabry factor is *G* = 0.179, then *α* = 3.095 and *β* = 1.862 or close [12]. Table 1 shows different values for the coil length and the result of the Fabry factor. The best value for length that gives a probe a (*G*) value equal to 0.17324 is 9.435 mm.

After determining the geometry of the coil, the next step was to determine the number of turns of the coil. First, we decided the maximum current input to the coil to be 0.457 Amps, so a copper wire with gauge AWG-25 satisfied the requirement. The input voltage was 5 V, and the frequency used was 28 kHz. For AWG-25, the wire diameter (*d*) was 0.45466 mm, which can be used to determine the number of turns for the coil. The core type used in the design was a ferrite core to focus the magnetic field, which increased the sensitivity of the coil for small-sized defect detection. Using Equation (4), the number of turns of the coil was calculated. In the equation, r1 is the inner radius of the coil, r2 is the outer radius of the coil, l is the coil length, and *d* is the diameter of the wire.

(4) $$\mathrm{Number}\;\mathrm{of}\;\mathrm{turns}=\frac{l}{d}\frac{r2-r1}{d}\times 0.8\;$$

**Figure 4:**
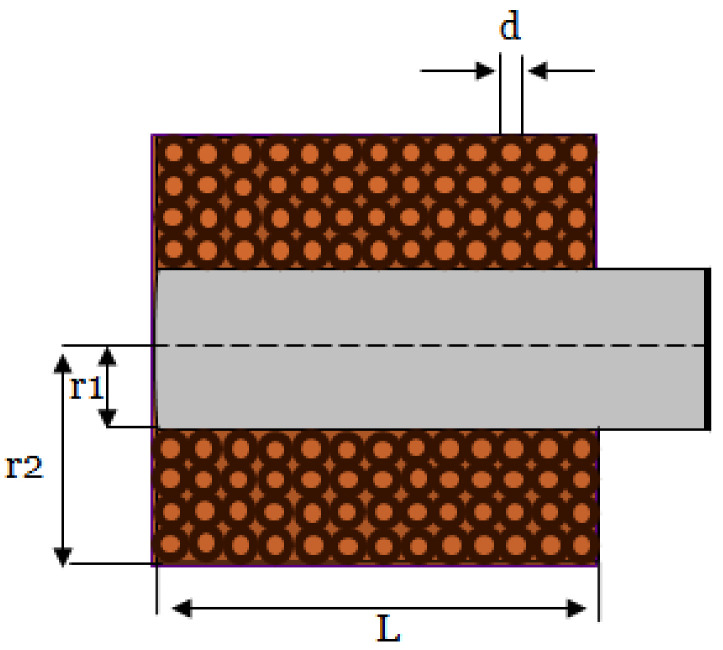
Cross-section of the coil geometry.

We took the fill factor into consideration, which was 0.8. After considering fabrication, the approximate number of turns was 164. An illustration of the coil geometry is shown in Figure 4. Equation (5) was used to calculate number of turns per layer.

(5) $$\mathrm{Number}\;\mathrm{of}\;\mathrm{turns}\;\mathrm{per}\;\mathrm{layer}=\frac{Length\;of\;the\;coil}{Diameter\;of\;the\;wire}$$

The number of turns per layer equaled approximately 20 turns per layer. The number of layers was calculated by dividing the number of turns by the number of turns per layer, which equals to approximately 8 layers. The total inductance calculated for the coil was 444.94 µH using Equation (6), where $\mu_{eff}$ is the effective permeability of the core [13]. The measured inductance value using the RLC meter was 518 µH.

(6) $$\mathrm{Inductance}\;\frac{\mu_{eff\times }(number\;of\;turns\;(r2+{r1))}^{2}}{127(13r2+9l-7r1)}$$

### 2.3. Tip Analysis and B_max Measurements

**Figure 5:**
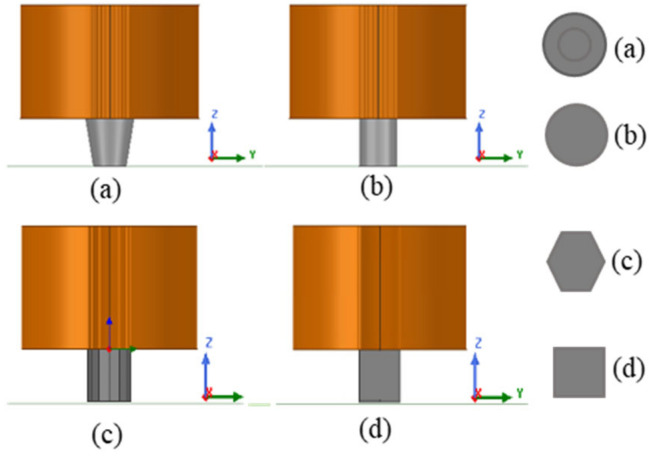
Different tip geometry, where (**a**) is a cone tip shape, (**b**) a cylindrical tip shape, (**c**) a polygon tip shape, and (**d**) a square tip shape.

As mentioned earlier, the probe works in the absolute mode, which means that only one coil is used to induce the eddy currents inside the material and sense the defect signal too. The probe had a core made of ferrite, which has a higher permeability compared to cores made of iron. The core length was 13.435 mm, where 9.435 mm is the same length as the length of the coil and 4 mm was considered for the core tip. Adding a tip to the designed coil helped focus the magnetic field and make it more confined. Another reason was to make it possible to detect very small defect sizes since there is a relationship between the defect size and the probe diameter. Different tip geometries, shown in Figure 5, were considered for the probe design, and the magnetic field (B_max) was calculated for each case. The distance between the surface of the tip and the substrate was fixed in all cases for consistency.

The value of the magnetic field (B_max) was measured at 4.2 mm distance under the coil in the case of having substrate under the coil and in the case of no substrate, as shown in Table 2. The material considered in all cases was stainless steel (316). Simulation was carried out using ANSYS Maxwell. The tip shapes considered for comparison were cylindrical, square, polygon, and cone shapes. The volume of all tips was kept constant for consistency. The volume of all the tips was around 153.9 mm^3^. The cylindrical tip shape showed a better B_max value under the probe, and it was selected for the probe design.

**Figure 6:**
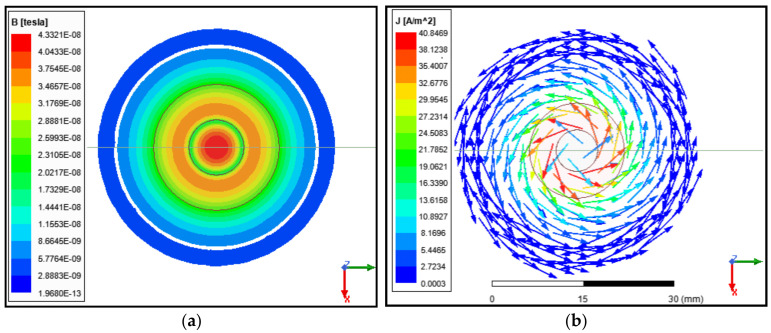
ANSYS eddy current simulation of the designed probe, where (**a**) shows the magnetic field distribution and (**b**) shows the eddy current distribution under the coil.

Adding a ferrite core focused the magnetic field to the center of the coil. As seen in Figure 6a, the highest B_max under the probe was around the center of the coil. As shown on the scale bar in Figure 6b, the eddy current distribution under the coil was almost double the size of the probe diameter. The probe diameter was 16 mm, meanwhile the distribution of the eddy currents was around 30 mm. This was one of the constraints of testing small size parts because of what is called the edge effect. The edge effect means that the edges of the part under test will give a false signal similar to a defect signal [14,15].

### 2.4. Model Design and Simulation on ANSYS Maxwell

**Figure 7:**
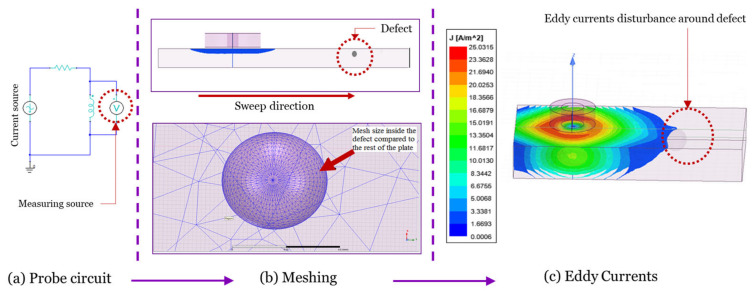
Graphical abstract of the simulation procedures.

After determining the coil dimensions and the probe design, a model with the same parameters was built on ANSYS. In Figure 7, a graphical abstract was shown to illustrate the simulation process.

**Figure 8:**
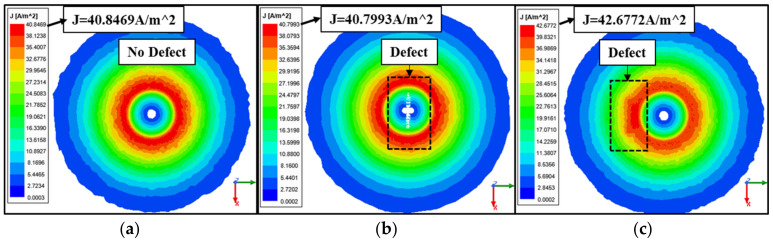
Magnitude of the eddy currents’ distribution, where (**a**) shows the eddy current distribution in the case that there is no defect, (**b**) shows the eddy current distribution if the defect is aligned with the center of the coil, and (**c**) shows the eddy current distribution if the defect is aligned with the coil winding.

The solution type used in the ANSYS Maxwell software tool version R2 was the eddy current solution type. For all types of designs and simulations, results were obtained after choosing different mesh sizes for a mesh dependency analysis. The mesh size was one of the main factors that affected the simulation results. Applying a fine mesh size with respect to the size of the defect will give better results, but of course, that will affect the simulation time a lot and cause the simulation to take a longer time and more CPU memory. The mesh type used for this model was the on-selection skin-depth-based, since it gave better simulation results when it comes to a very small size defect compared to the on-selection length-based one. The surface triangle length was 0.3 mm. The sweep over frequency was carried out over a big range of frequencies (10 kHz–500 kHz) to find the best frequency value that gives better results for the designed probe. The frequency value chosen for the designed probe was 28 kHz. Regarding the AC current used in the simulation, it was produced using a circuit that was created using an ANSYS circuit, which was attached to the coil terminal and the winding. It is a small RL circuit, which is shown in Figure 7. Building this circuit and attaching it to the winding of the coil helped in measuring the impedance of the coil. As shown in Figure 8, three different cases were considered for simulation. The first case was where there was no defect added to the stainless steel plate; the maximum value for the current density was 40.8469 A/m^2^. For the second case, a defect was added to the center of the plate where it was aligned with the center of the coil to see the effect of the defect on the current density value. The current density value for this case was 40.7993 A/m^2^, which was less than the case where there was no defect added to the plate.

The third case considered was adding the defect to the plate in a way that it was aligned with the coil winding to see if the maximum current density value was different from case two, where the defect was aligned with the center of the coil. The maximum value of the current density was 42.6772 A/m^2^ which was higher than the other two cases.

## 3. Measurement Results and Discussion

To test the designed probe, three different cases were considered for testing machined stainless steel plates with defects that acted as mock-ups of parts made by additive manufacturing. The tests were designed to detect notches with the same width but different lengths, notches with different widths and the same length, and finally blind holes with different sizes. In [16], bigger blind holes sizes were detected. A comparison was made between the response of the designed probe in the experiment and the analytical and simulation results. The types of defects considered for all the above cases are subsurface blind holes which simulate pores, and subsurface notches which simulate the cracks created between the layers of the additively manufactured parts. The blind hole sizes were 0.17 mm, 0.2 mm, 0.27 mm, and 0.3 mm in radius. The sizes for the notches were 0.437 mm, 0.47 mm, and 0.57 mm in width, with lengths of 5 mm, 15 mm, and 25.2 mm. All tests were carried out from the opposite side of the defect to simulate subsurface defects.

### 3.1. Probe Impedance Calculation

**Figure 9:**
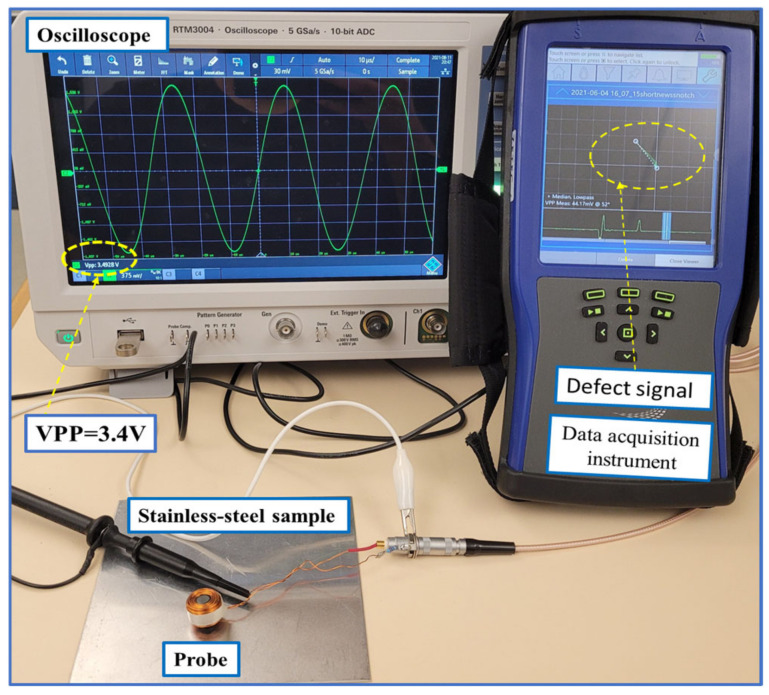
Experimental setup of the detection process.

Measuring the impedance of the probe will help determine the location of flaws inside the parts under test. If a flaw exists inside the material, it causes a change in the probe impedance. In all the following three cases, probe impedance was calculated for the case of having a plate made of stainless steel. An experimental setup of the detection process is shown in Figure 9. The measured peak-to-peak voltage identified using the oscilloscope was 3.4 V and is the voltage measured at the probe terminal on top of the stainless steel plate. A resistance of 27 ohms was connected in series with the coil. The input peak-to-peak voltage to the coil was 5 V. The RMS value of the input AC current measured using a current probe was 0.0148 Amps. In Figure 9, the measured defect signal is shown at the data acquisition instrument impedance plane. The measured defect signal is represented as a measured peak-to-peak voltage at the coil terminal. The total impedance of the probe is the measured coil impedance in free space $Z_{FS}$ added to the coil impedance on top of material $Z_{m}$:

(7) $$\;{Z=Z_{FS}+Z}_{m}$$

Using the measurements obtained from the experimental setup, the impedance of the coil (Z) on top of the stainless steel plate was calculated as shown below:

(8) $$Z=\sqrt{R^{2}+{x_{l}}^{2}}=\frac{V}{I}$$

where R is the resistance and $x_{l}$ is the inductive reactance, I is the current, the peak-to-peak voltage (*Vpp*) is 3.4 V and the RMS value of it s 1.202 V. So, the measured impedance value of the coil using Equation (8) was 1.202/0.0148 = 81.2 Ω. In analytical modeling, the general expression for the change of coil impedance on top of a multi-layer structure is derived using Equation (9).

(9) $$\Delta Z=-j2\omega \pi {ϻ}_{0\;}b^{5}\sum_{m=1}^{\infty }{{L_{Nm}C}_{m}}^{2}exp(-2x_{m}l)$$

where the equation for $C_{m\;}$shown below is also called the coil factor. It can only be determined by the inner radius ($r_{1}$), outer radius ($r_{2}$), and the height of the coil (h). Also, $J_{1}(x)$ is a first-order Bessel function [17]. The impedance value obtained analytically using Equation (9) is 79.8 ohm, which almost matches the value obtained experimentally using Equation (8). The impedance of the coil obtained from the simulation on top of a 2 mm thick plate without defect was 90.8 ohm. The complex function ($L_{Nm}$) can be determined by the plate thickness, the material relative permeability, and electrical conductivity. The lift-off distance is (l) and the truncated field region is (*b*). Also, ω is the angular frequency, and ${ϻ}_{0}$ is the permeability of free space.

The change in the coil impedance in case of a defect is calculated based on the vector potential in Equation (10) [18].

(10) $$z=\left[\frac{3}{2}\sigma \omega^{2}{\left(\frac{A}{I}\right)}^{2}\right]\cdot Vol\alpha_{22}$$

where A is the vector potential, σ is the electrical conductivity, and ω is the angular frequency. The second part, which is $Vol\alpha_{22}$, depends on the defect’s geometry. The final formula shown in Equation (10) is based on the Dodd and Deeds model that gives a closed integral form solution to the vector potential [19]. Other methods were used to solve the vector potential, such as the Truncated Region Eigen-function expansion method (TREE) mentioned in [20]. Usually, the solution is expressed as an integral form based on the Fourier or the Bessel integral. This method is used to truncate the solution domain of the problem and the modified solution obtained is in a series expansion form instead of integral form. The truncated region method was the one used in this paper. Cauchy’s argument method was used to calculate the complex eigenvalues, which satisfied the boundary conditions between the defect area and the material [21]. Numerical modeling of general cracks from the viewpoint of eddy current simulations on the specimens made of stainless steel 316 is shown in [22].

### 3.2. Case 1: Three Notches Same Width Different Length

**Figure 10:**
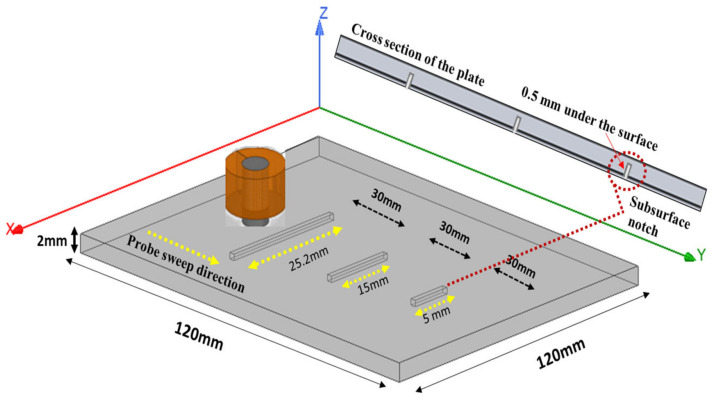
Plate with three notches of same the width and different lengths.

Artificial defects were seeded in stainless steel plates to assess the feasibility of detecting various flaws with different widths and lengths. The first test for the designed probe was to detect three notches with widths of 0.43 mm and lengths of 5 mm, 15 mm, and 25.2 mm. Notches were 0.5 mm from the surface of the plate, and the thickness of the plate was 2 mm, as shown in Figure 10. Detection was carried out from the opposite side of the notches to simulate subsurface cracks inside the material. Scanning was carried out across the notch in the (Y) direction. Results were compared to the simulation model that was created using ANSYS Maxwell and analytical modeling. The samples were made of stainless steel (316) with a conductivity of 1.33 Ms and relative permeability of 1.01.

**Figure 11:**
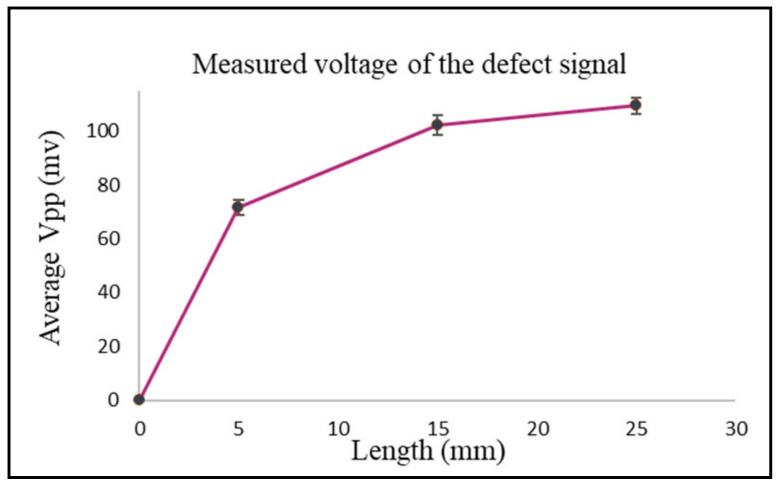
The average and standard deviation values of the defect for each length.

**Figure 12:**
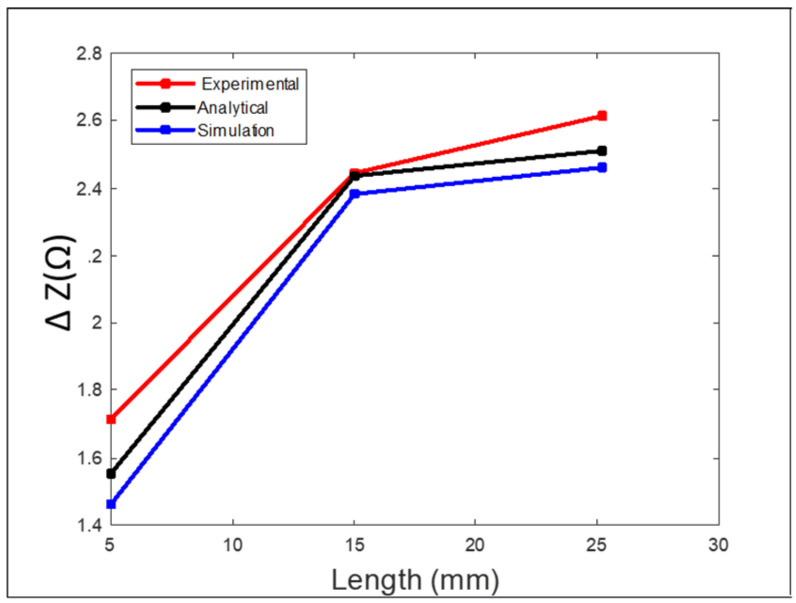
Comparison between the experimental, analytical, and simulation results for case 1.

Figure 11 shows the measured peak-to-peak voltage value for each defect. To get better and accurate results, a total of six readings were taken for each defect. The probe sweep direction with respect to the location of the defects is shown in Figure 10. The standard deviation and the average of all readings was calculated. During the experiment, it was noticed that the best way to get accurate results for each defect was to not change the lift-off distance between the probe and the specimen. To achieve that, it was better to sweep the probe back and forth on top of the defect to get multiple readings with the same phase, which helped improve the standard deviation values for each defect. The RMS values of the average measured peak-to-peak voltage of each defect were the ones used in Figure 12, where they are represented in terms of impedance (ΔZ).

The values obtained from the experimental results were higher than the ones obtained from the simulation and analytical modeling. The longer the length of the defect, the greater the change in the coil impedance. At a defect length of 25.2 mm, there was a higher value representing the coil impedance change compared to the values obtained at defect lengths of 15 mm and 5 mm. For defect lengths of 15 mm and 25 mm the simulation results were better since the mesh size used in the simulation was suitable and big enough compared to the defect size, which gave more accurate results. Several simulation models were run with different mesh sizes for mesh analysis. In cases of small defect sizes, the mesh size had to be very small to get better simulation results, which requires more computational power and a longer simulation time. More elaboration can be seen in Section 3.4 for all experiments.

### 3.3. Case 2: Three Notches of the Same Length and Different Widths

**Figure 13:**
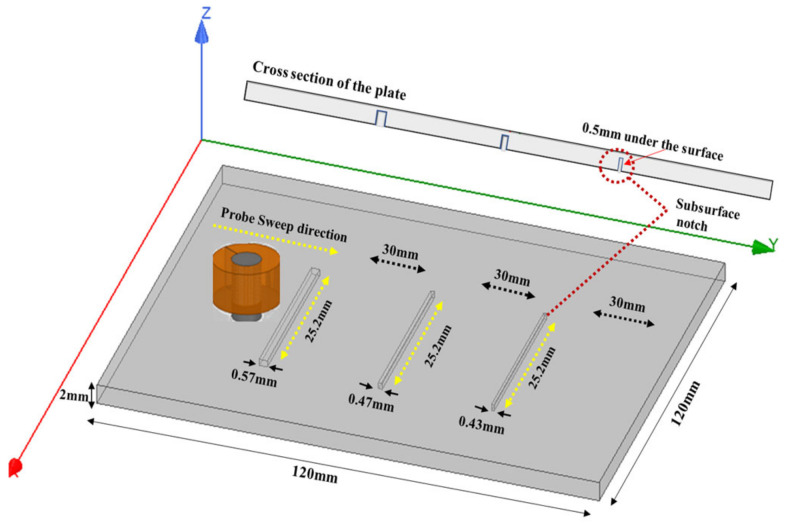
Plate with three notches of the same length and different widths.

The test for the designed probe was to detect three notches with 0.57 mm, 0.47 mm, and 0.437 mm widths. The three notches all had the same length of 25.2 mm, as shown in Figure 13. Notches were 0.5 mm from the surface of the plate, and the thickness of the plate was 2 mm. Detection was carried out from the opposite side of the notches to simulate subsurface cracks. Scanning was carried out across the notches in the (Y) direction. Results were compared to the simulation model that was created using ANSYS Maxwell and analytical modeling.

**Figure 14:**
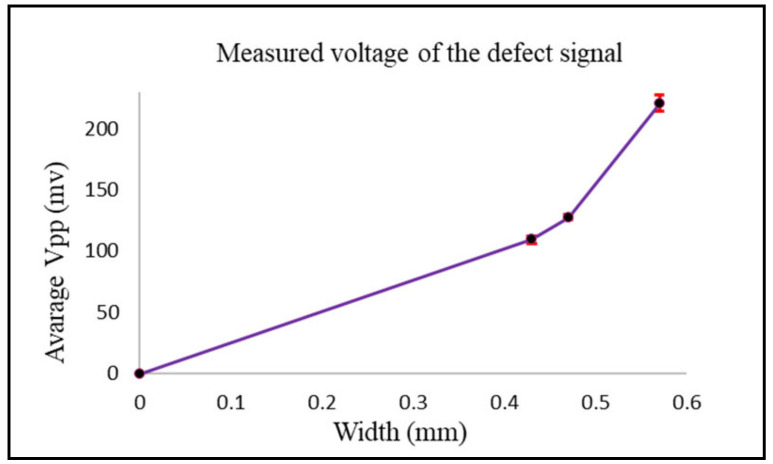
The average and standard deviation values of the defect for each width.

**Figure 15:**
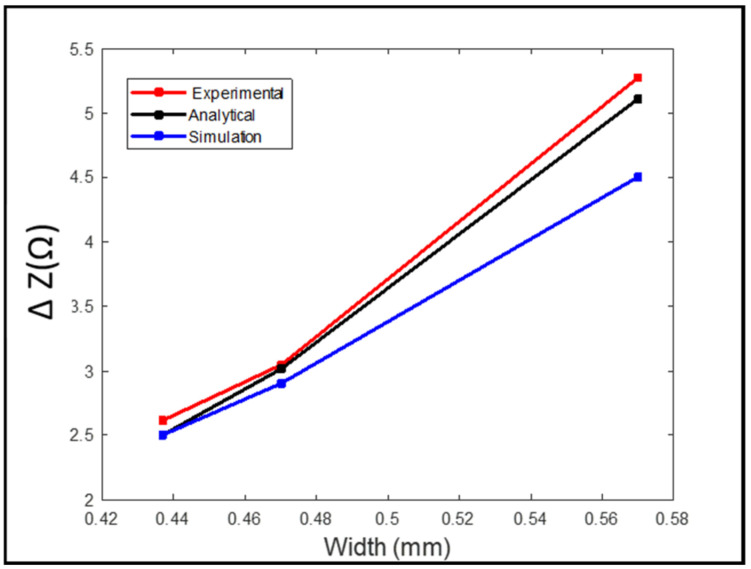
Comparison between the experimental, analytical, and simulation results for case 2.

Figure 14 shows the measured peak-to-peak voltage value for each defect. To get better and accurate results, a total of six readings were taken for each defect. The probe sweep direction with respect to the location of the defects is shown in Figure 13. The standard deviation and the average of all readings were calculated. The RMS values of the average measured peak-to-peak voltage of each defect were the ones used in Figure 15, where they are represented in terms of impedance (ΔZ).

The values obtained from the simulation were lower than the ones obtained from the analytical and experimental results. The higher the width of the defect, the higher the change in the coil impedance. At a defect width of 0.57 mm, there was a higher value representing the coil impedance change compared to the values obtained from the defects with widths of 0.47 mm and 0.43 mm. Also, there was no big difference in the values obtained from the 0.47 mm defect width compared to the ones obtained from the 0.43 mm defect width since there was no big difference between both widths.

### 3.4. Case 3: Blind Holes with Different Diameters

**Figure 16:**
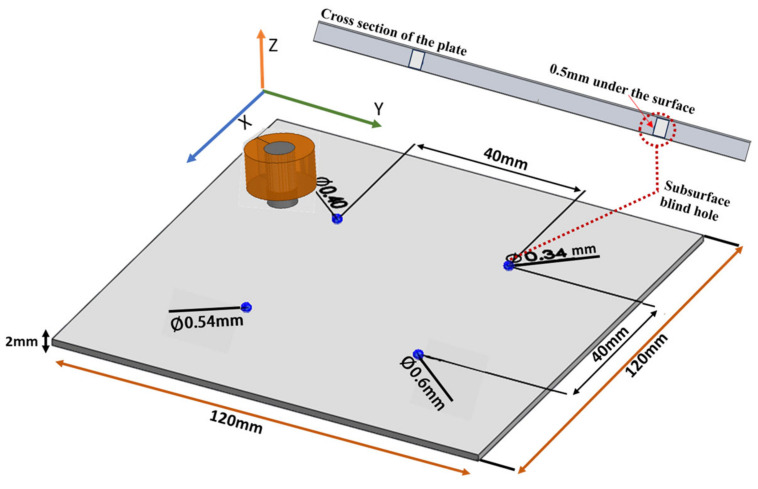
Plate with blind holes with different diameters.

The test for the designed probe was to detect four blind holes with diameters of 0.34 mm, 0.4 mm, 0.54 mm, and 0.6 mm. The four blind holes were 0.5 mm from the surface of the plate and had a depth of 1.5 mm. The plate thickness was 2 mm, as shown in Figure 16. Detection was carried out from the opposite side of the blind holes to simulate subsurface pores inside the material. Results were compared to the simulation model that was created using ANSYS Maxwell and analytical modeling.

**Figure 17:**
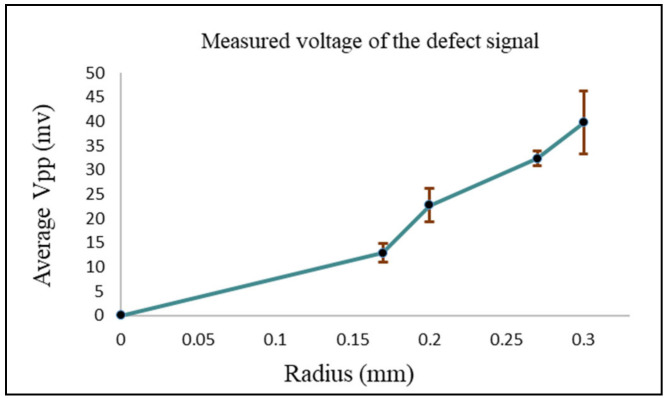
The average and standard deviation values of the defect for each void radius.

**Figure 18:**
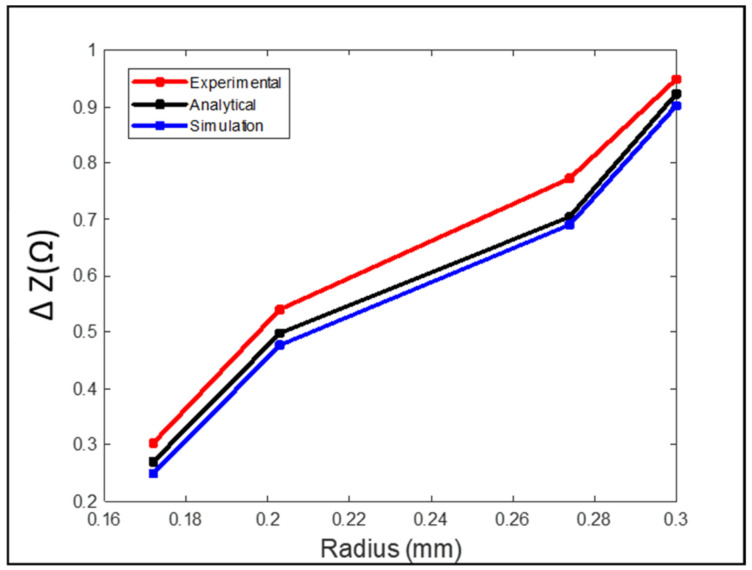
Comparison between the experimental, analytical, and simulation results for case 3.

Figure 17 shows the measured peak-to-peak voltage value for each defect. To get better and accurate results, multiple readings were taken for each defect. The standard deviation and the average of all readings were calculated. The RMS values of the average measured peak-to-peak voltage of each defect were the ones used in Figure 18, where they are represented in terms of impedance (ΔZ).

In Figure 18, there is a match between the analytical and experimental results compared to simulation results, which are a bit lower. The mesh size used for the simulation was not small enough compared to the blind hole diameter, which played a big role in the accuracy of the results. The smaller the diameter of the blind hole, the smaller the probe’s response was to it. In all the above cases, the simulation values for each case were less than the experimental results. The reason is that the accuracy of the simulation results was affected by the mesh size and the boundary conditions. Since the size of the blind holes is very small, the mesh size must be small enough to get a better result, and that consumes a lot of time and memory. The simulation results for the notches cases were better since it was possible to increase the mesh size. By minimizing the energy error, the mesh size can be chosen in a way that keeps the balance between computing resource usage and the accuracy of the results. The measured signal value during the experiment was always higher in all cases. For the simulation and analytical modeling, the conditions were ideal compared to the experiment, which affected the accuracy of the measured defect signal. The probe was better able to detect defects on the samples with smooth surfaces compared to the samples with high surface roughness. Machining could be carried out on samples with a surface roughness of higher than 2 µm to smooth its surface in order to suppress the noise signal of the rough surface for better defect detection. Rough surfaces may produce a signal that is similar to the signal obtained from small size defects, which makes the detection process difficult [23,24,25].

### 3.5. Testing with AM Samples

In this section different cases are considered for a sensitivity analysis during running the experiment. All parts in this section were made by laser powder bed fusion technology (LPBF). The cases are as follows:

Testing samples made by LPBF with defects close to the edge to find the best way to detect those defects without getting a false signal produced by the sample’s edges that represent a crack. The second case explores the effect of different lift-off distances on the accuracy of defect detection. The third case explores detecting multiple defects that exist in the same region or defects that are separated by a small distance in between. All testing was carried out from the opposite side of the defects.

#### 3.5.1. Testing Close to an Edge

**Figure 19:**
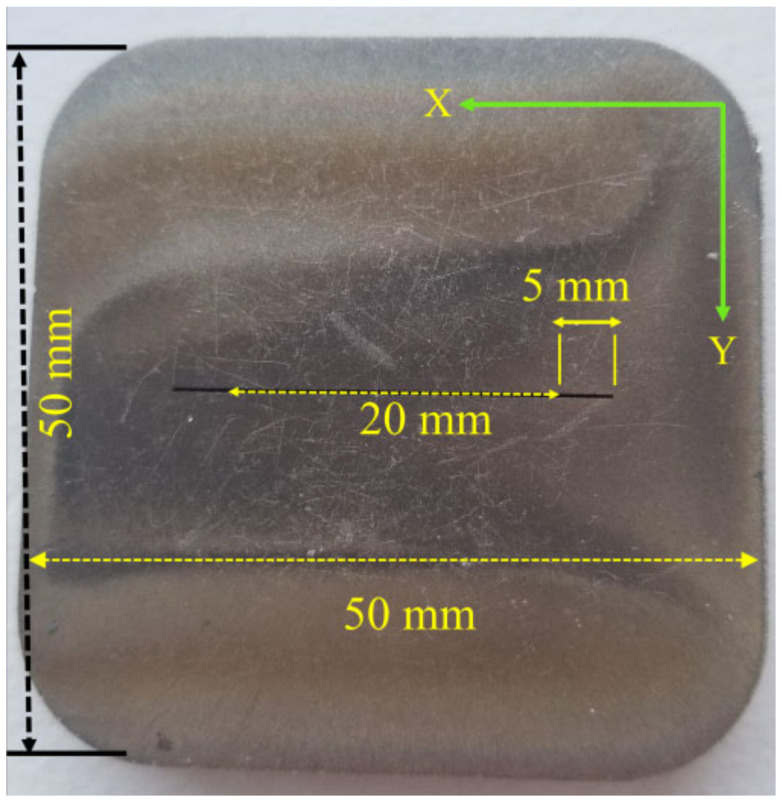
Notches close to edges in stainless steel samples.

The edge effect is one of the challenges when it comes to detecting flaws in small samples. The edges of the samples will produce a false signal that looks like a signal produced by a crack. The best way to avoid the effect of edges is to move the probe parallel to the edge. Figure 19 shows the dimensions of a stainless steel sample that has two notches close to an edge. Instead of moving the probe in the X direction to detect the defect, it is better to move it in the Y direction parallel to the edge of the sample, that way the effect of edges will be eliminated. The probe was moved on top of the stainless steel (SS316L) sample from the opposite direction of the defect to simulate subsurface defects. The plate used during the experiment had a 0.1 mm notch width. The average measured voltage at the probe terminal was 54.11 mV.

#### 3.5.2. Effect of Lift-Off Distance

The same sample used in Figure 19 was used in the second case as well to show the effect of having different lift-off distances between the probe and the samples. Two lift-off distances were considered during the experiment. The first lift-off distance considered was 1 mm and this was carried out by adding a sheet made of plastic with 1 mm thickness between the probe and the stainless steel plate. The plastic sheet had zero conductivity. The second lift-off distance considered was 2 mm and this was carried out by adding a sheet made of plastic with 2 mm thickness between the probe and the stainless steel plate.

The higher the lift-off distance between the probe and the part under test, the less eddy currents are produced inside the material, which leads to a smaller standard depth of penetration. One more thing noticed during the experiment was that the effect of surface roughness was suppressed by adding a sheet made of plastic or any other non-conductive material between the probe and the test piece. The plate used during the experiment had a 0.1 mm notch width. The average measured voltage at the probe terminal was 54.11 mV. In the case of a 1 mm lift-off distance, the measured peak-to peak voltage value at the probe terminal was less, at around 42.7 mV. In the case of a 2 mm lift-off distance, the measured peak-to-peak voltage value at the probe terminal was very weak, at around 35.03 mV.

#### 3.5.3. Multiple Defects in the Same Region

**Figure 20:**
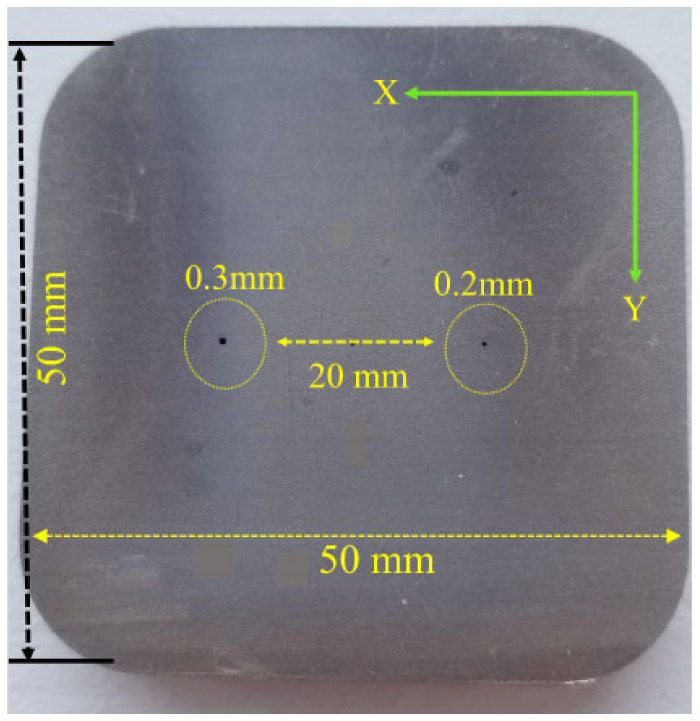
Two voids separated by a very small distance in a stainless steel sample.

**Figure 21:**
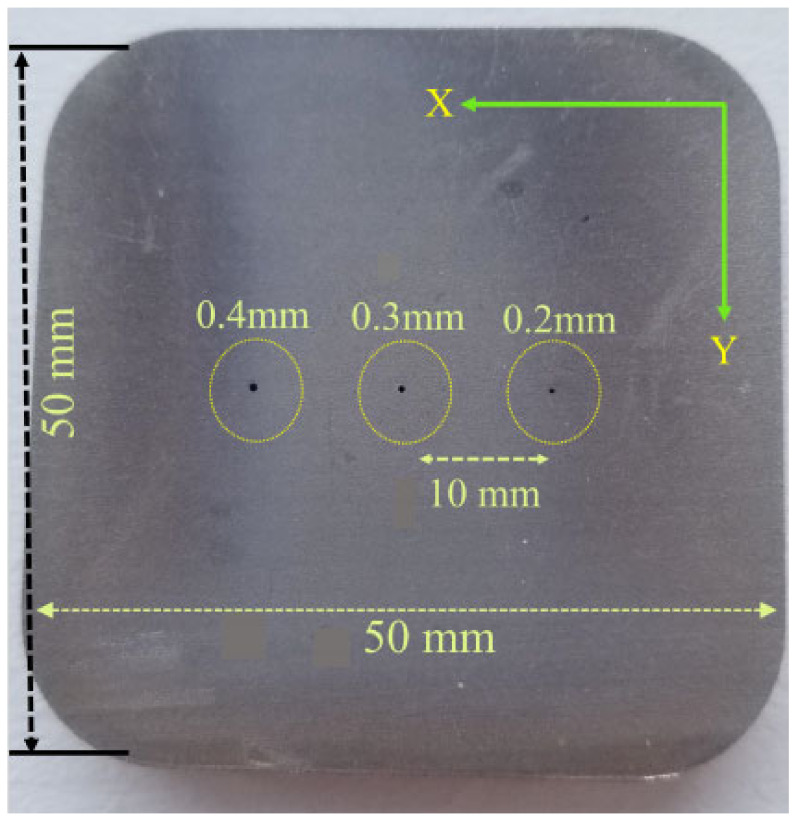
Three voids separated by a very small distance in a stainless steel sample.

All previous testing was carried out on samples that had only one defect inside the material. In this section other cases were considered, where the detection was carried out on samples that had multiple defects in the same region as other defects that were separated by a very small distance to see the effect of them on the detected defect signal. Samples used during the experiment are shown in Figure 20 and Figure 21. Testing was carried out from the opposite side of the defects to represent a subsurface defect.

Figure 20 shows a stainless steel plate that has two defects separated by 20 mm distance. The diameter of the designed probe was 16 mm, which was almost as big as the distance between both voids. The eddy currents created in the material under the probe were usually around twice the size of the probe diameter. The probe was moved in the X direction on top of the stainless steel sample. Since the probe diameter was around 16 mm, the eddy currents created under it had around a 30 mm diameter circular loop. The distance between the two voids was too close compared to the size of the eddy current’s circular loop diameter, which eventually showed only one peak value for the measured voltage for both defects instead of showing a different peak value for each defect. The average measured peak-to-peak voltage value of both defects together was 782.22 mV. Moving the probe on top of the 0.2 mm defect only gave a value of 264.03 mV. Moving the probe on top of the 0.3 mm defect only gave a value of 314.6 mV.

Figure 21 shows a stainless steel plate that has three defects separated by a 10 mm distance.

The short distance between the defects in the case of moving the probe in the X direction showed only one peak change in the measured voltage of the probe instead of three different peaks, one for each defect. The average measured peak-to-peak voltage value of the three defects together was 1175.74 mV. Moving the probe on top of the 0.2 mm defect only gave a value of 264.03 mV. Moving the probe on top of the 0.3 mm defect only gave a value of 314.6 mV. Moving the probe on top of the 0.4 mm defect only gave a value of 410.28 mV. Results are shown in Table 3. If the distance between the defects is smaller than the diameter of the probe the issue is as follows: the eddy current’s distribution under the probe is usually almost twice the diameter of the probe and will be affected if there are multiple defects in the same region, and as a result, it will give a signal with a higher voltage value that represents the voltage measured from all the defects combined. The geometry of the defect and the location of the defect inside the material has a strong effect on the measured voltage value. A bigger defect size results in cutting more eddy current flow. Since the notch in Section 3.5.2 has a bigger size than the hole size in Figure 17, depending on the sweep direction of the probe, it will result in cutting more eddy current lines and that will result in measuring higher voltage values at the probe terminals.

The core type used in the design was a ferrite core, which focused the magnetic field mainly under the probe, which increased the sensitivity of the probe for small size defect detection. Looking at Figure 8, the eddy current distribution was almost twice the diameter of the probe, but the secondary magnetic field produced by the eddy currents were mainly concentrated under the core of the probe, which is shown in the red area. By moving the probe in the Y direction as shown in Figure 20 and Figure 21, and since the defect was right under the core of the probe where the magnetic field was mainly concentrated, it had a high impact on the measured voltage. Any defects that exist towards the outer loops of the eddy currents will not affect the measured voltage at the probe terminals regardless of their distance from the center of the probe. Therefore, for this reason the produced voltages for the holes 0.2 mm and 0.3 mm seemed identical. The accuracy of defect sizing (length, depth, and width) is highly dependent on the resolution of the instrument and the skill of the operator. Typically, sizing accuracy can be within ±10% of the actual defect size under controlled conditions. A total of six readings were taken for each defect and the overall accuracy percentage was 88.03%.

## 4. Conclusions

In this paper, the main steps in designing an eddy current probe were carried out, specifically addressing challenges encountered in the NDT of parts made by additive manufacturing, such as surface roughness. A critical aspect of the probe’s design is the inclusion of a bottom surface coating aimed at smoothing out the measured defect signals from any ripples, thereby enhancing accuracy. Sensitivity to small flaws is paramount, necessitating careful consideration of coil dimensions relative to detectable defect sizes. A smaller coil diameter should be considered for better detection for small defect sizes. Presently, the current probe design can detect blind holes within the range of 0.3 mm to 0.6 mm diameter. Also, it can detect cracks with different lengths in the range of 0.43 mm to 0.6 mm width. Having a cylindrical tip shape allowed for focusing the magnetic field to the center of the coil. However, when probing for defects from the untipped side, blind holes of 0.17 mm and 0.2 mm were undetectable. The tip’s function in focusing the magnetic field and eddy currents beneath the coil renders these small-diameter blind holes disruptive to detection. The probe was tested on stainless steel (SS316L) samples made by laser powder bed fusion technology. The probe detected different subsurface defect types with different sizes. The smallest subsurface void size detected had a diameter of 0.2 mm. Also, a 0.1 mm subsurface notch width was detected successfully. In future work, the probe will be tested on other samples with smaller defect sizes at different depths and with varying degrees of surface roughness.

## Figures and Tables

**Table 1:** Different values for the Fabry factor (*G*) at different coil lengths for fixed inner and outer diameters of 7 mm and 16 mm, respectively.

Length	Alpha	Beta	G
5	2.28	0.71429	0.1529
9.435	2.28	1.3479	0.17324
20.76	2.28	2.9657	0.15885
31.14	2.28	4.4486	0.13905
41.52	2.28	5.9314	0.12376
51.9	2.28	7.4143	0.1121
62.28	2.28	8.8971	0.10318

**Table 2:** B_max value for each tip shape at a 4.2 mm distance under the coil.

Tip Shape	No Plate under the Coil	In Case of a Plate under the Coil
Cylindrical	B_max=1.45 $\times \;10^{-8}$ T	$B\_\max=4.3\times \;10^{-8}$ T
Cone	$B\_\max=1.23\;\times \;10^{-8}$ T	$B\_\max=3.81\;\times \;10^{-8}$ T
Polygon	$B\_\max=1.39\;\times \;10^{-8}$ T	$B\_\max=4.01\;\times \;10^{-8}$ T
Rectangular	$B\_\max=1.201\;\times \;10^{-8}$ T	$B\_\max=3.78\;\times \;10^{-8}$ T

**Table 3:** Measured voltage of the probe for cases of multiple defects in the same region.

Defect Size	Path of Travel	Measured Voltage
0.2 mm	Y direction	264.03 mV
0.3 mm	Y direction	314.6 mV
0.4 mm	Y direction	410.28 mV
Both defects (0.2 mm, 0.3 mm)	X direction	782.22 mV
All defects (0.2 mm, 0.3 mm, 0.4 mm)	X direction	1175.74 mV

## Data Availability

Data are contained within the article.
